# Clinical significance of CD24 as a predictor of bladder cancer recurrence

**DOI:** 10.3892/ol.2013.1357

**Published:** 2013-05-21

**Authors:** CHUNXIAO LIU, SHAOBO ZHENG, HAIYAN SHEN, KAI XU, JIE CHEN, HULIN LI, YAWEN XU, ABAI XU, BINSHEN CHEN, HARUKI KAKU, YASUTOMO NASU, HIROMI KUMON, PENG HUANG, MASAMI WATANABE

**Affiliations:** 1 Department of Urology, Zhujiang Hospital, Southern Medical University, Haizhu, Guangzhou, Guangdong, P.R. China;; 2 Center for Innovative Clinical Medicine, Okayama University Hospital, Okayama, Japan; 3 Department of Urology, Okayama University Graduate School of Medicine, Dentistry and Pharmaceutical Sciences, Okayama, Japan

**Keywords:** CD24, bladder cancer, immunohistochemistry, tumor, recurrence

## Abstract

Cluster of differentiation (CD)24 was originally described as a B lymphocyte marker and has recently received considerable attention in cancer research as its overexpression has been observed in several types of carcinoma. The CD24 molecule is a glycosyl-phosphatidylinositol-linked cell surface protein that appears to be associated with aggressive cancers involving invasion and metastasis. However, the expression of CD24 in human bladder cancer and its clinical significance remains largely unknown and no association has been reported between CD24 overexpression and human bladder tumor recurrence. In the present study, the CD24 expression in cancer tissues obtained during transurethral surgery and the subsequent intra-bladder tumor recurrence following surgery were assessed. Immunohistochemical staining was performed and the intensity of CD24 staining was semi-quantitatively evaluated. CD24 expression was observed more frequently in high-grade bladder tumors (G2–G3) than low-grade tumors (G1). Positive CD24 expression was significantly associated with intra-bladder tumor recurrence following surgery and increased staining intensity was also correlated with recurrence. The positive association between CD24 expression and tumor recurrence was observed in each tumor category (stages Ta and T1, low and high grade). The results demonstrated that CD24 expression is significantly associated with bladder tumor recurrence. To the best of our knowledge, this is the first study to reveal the significance of CD24 as a predictor of bladder cancer recurrence. These insights may lead to future therapeutic strategies targeting CD24 to prevent the dissemination of bladder cancer cells and tumor recurrence.

## Introduction

Bladder cancer is the second most common urological malignancy and represents a significant cause of morbidity and mortality worldwide (1). The disease consists of two principal forms of cancer, superficial and invasive, with the majority of bladder carcinomas as the former type at the time of diagnosis. Superficial cancers exhibit papillary and multifocal tumor growth, which often recurs following transurethral surgery and occasionally progresses to become an invasive disease. By contrast, invasive cancer is usually nodular, metastasizes to distant organs during the early phase of the disease and has a poor prognosis. The treatment for these cancers usually involves transurethral resection of the bladder tumors or a combination of chemotherapy, immunotherapy and radical cystectomy. However, a significant number of patients suffer from disease recurrence and progression. Therefore, a greater understanding of the molecular characteristics involved in bladder cancer recurrence is required to develop improved, more effective treatments.

Cluster of differentiation (CD)24 is a small, heavily glycosylated, mucin-like cell surface protein (27 amino acids long) that binds to the membrane via a glycosyl-phosphatidylinositol anchor (2). CD24 molecules are expressed in hematopoietic cells, such as B lymphocytes and neutrophils, and in certain epithelial cells, including keratinocytes and renal tubular epithelial cells (2,3). It has been reported that CD24 is important in cell selection and maturation for specific cellular functions (4). CD24 has also been reported to be a ligand for P-selectin, an adhesion receptor on activated endothelial cells and platelets (5,6), suggesting that the molecule functionally enhances the metastatic potential of cancer cells. CD24 has recently received attention in tumor biology research, as several studies have reported that the protein is broadly overexpressed in numerous types of cancer cell from the lung, breast, prostate, liver, kidney, pancreas and ovary, as well as in lymphomas (3,4). These studies demonstrated that CD24 overexpression was markedly associated with a more aggressive course of the disease. However, the correlation of CD24 expression with bladder cancer and its prognostic significance remain largely unknown.

The present study evaluated the CD24 expression in resected tumor specimens of bladder cancer and analyzed the correlation between this expression and the clinicopathological parameters. Since the association between CD24 overexpression and bladder cancer recurrence has not been reported previously, the study was focused on the CD24 expression in cancer tissues and the intra-bladder tumor recurrence following transurethral surgical resection.

## Materials and methods

### Patients and tissue samples

The present study included 125 patients with primary superficial bladder cancer (Ta and T1) who were treated in the Department of Urology, Zhujiang Hospital of Southern Medical University (Guangzhou, China) between 2005 and 2008. Cases of muscle invasive cancer (T2, T3 and T4), carcinoma *in situ* (CIS) or diseases other than urothelial carcinoma were excluded from the study and none of the patients included in the analysis had received pre-operative treatment. All patients were classified according to the 1997 UICC TNM classification for pathological staging and the 1973 World Health Organization classification for pathological grading. The bladder tumor lesions of the 125 patients were treated with transurethral resection of bladder tumor (TUR-Bt) surgery, and the paraffin-embedded samples were prepared for hematoxylin and eosin (HE) staining and immunohistochemical examination. Follow-up examinations were conducted cystoscopically to detect any tumor recurrence for three to six months following the transurethral surgery.

This study was approved by the ethics committee of Zhujiang Hospital of Southern Medical University, Haizhu, Guangzhou, Guangdong, China. Written informed consent was obtained from the patients.

### Immunohistochemical staining of CD24

The bladder cancer tissue specimens were fixed in 10% neutral buffered formalin and subsequently embedded in paraffin. The specimens were sectioned to a thickness of 4.5 *μ*m, then de-paraffinized with xylene and rehydrated for further staining with HE or immunohistochemistry. The immunohistochemical staining for the CD24 expression was performed as described previously (7,8). Briefly, the endogenous peroxidase activity in the rehydrated sections was blocked with 3% H_2_O_2_ and the antigen was retrieved by heat treatment for 30 min in 10 mmol/l citrate buffer. The sections were incubated for 10 min in 10% normal goat serum, then incubated overnight at 4°C with a primary rabbit polyclonal antibody against the CD24 antigen (1:100 dilution, ab110448; Abcam, Cambridge, MA, USA). The tissue sections were then incubated at room temperature for 1 h with anti-rabbit peroxidase-conjugated secondary antibody. The bound antibodies were detected using diaminobenzidine-tetrahydrochloride as the substrate and the sections were counterstained with hematoxylin. The sections were then dehydrated, mounted and observed using light microscopy.

### Immunohistochemical analysis of CD24 expression

The sections immunostained for CD24 were evaluated by two independent pathologists or urologists who were blinded to the clinicopathological data and clinical outcomes of the patients. CD24 expression was assessed by calculating the percentage of tumor cells that showed immunoreactivity in the microscopic field and then classified as negative (∼1%) or positive (>1%) for each tumor specimen (9). The number of positively-stained tumor cells showing immunoreactivity in the cytoplasm and the number of negatively-stained cells were counted in 10 representative microscopic fields. The percentage of positive cells was then calculated. In addition, the staining intensity was determined as reported previously (10), where the degree of intensity was classified into three categories; weak, moderate and strong. The most frequently recorded staining intensity of the 10 representative microscopic fields was determined as the intensity of the tumor sections. The results of these assessments were obtained from the two researchers and compared. Any discrepancies were resolved by reassessment of the sections by the two researchers until a consensus was reached.

### Statistical analysis

The SPSS version 17.0 software program (SPSS Inc., Chicago, IL, USA) was used for the statistical analysis. The data are presented as the mean ± standard error. Fisher’s exact test was used to evaluate the association between the CD24 expression and the clinicopathological parameters. The recurrence-free rate of bladder cancer following transurethral surgery was estimated based on the Kaplan-Meier method. P<0.05 was considered to indicate a statistically significant difference.

## Results

### Clinicopathological characteristics

The clinicopathological characteristics of the bladder cancer patients are shown in Table I. A total of 125 patients with an average age 61.4 years (range, 18–88 years) were enrolled in the present study. The pathological stages were Ta (intramucosal, n=71) or T1 (submucosal invasive, n=54) and the cancer grade was either G1 (n=29), G2 (n=62) or G3 (n=34). Bladder cancer recurrence developed in 67 (53.6%) of the 125 patients, as detected by cystoscopic examination following transurethral surgery. Disease recurrence was defined as any evidence of a tumor in the bladder at least three months subsequent to treatment.

### CD24 expression and cellular distribution

**Figure 1.**
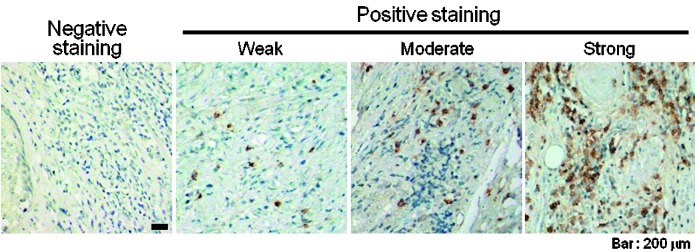
CD24 expression in human bladder cancer tissues. Immunohistochemical staining was performed and representative cases, including cases with positive (weak, moderate and strong) and negative expression, are shown. CD24, cluster of differentiation-24.

The expression and cellular distribution of the CD24 protein were determined by immunohistochemical staining in 125 paraffin-embedded bladder cancer tissues. Several specimens of adjacent normal bladder tissues were included in these paraffin blocks for comparison. The CD24 expression was typically negative in the normal urothelial cells of the bladder epithelium (data not shown). The specific signals of the CD24 protein were localized mainly in the cytoplasm of the cancer cells and were indicated by brown staining (Fig. 1). The CD24 protein expression was positive in 79 (63.2%) of the bladder cancer cases and the representative staining pattern [negative or positive (weak, moderate or strong)] is shown in Fig. 1.

### Association of CD24 expression with clinicopathological parameters of bladder cancer

CD24 immunoreactivity was positively correlated with certain clinicopathological parameters (Table II). There was a significant association between CD24 expression and the cancer grade and the recurrence of the bladder tumors [low-grade (G1, 8 out of 29, 27.6%) and high-grade (G2–G3, 71 out of 96, 74.0%); recurrence-negative (22 out of 58, 37.9%) and recurrence-positive (57 out of 67, 85.1%)].

### Correlation between CD24 expression and bladder cancer recurrence-free rate

**Figure 2.**
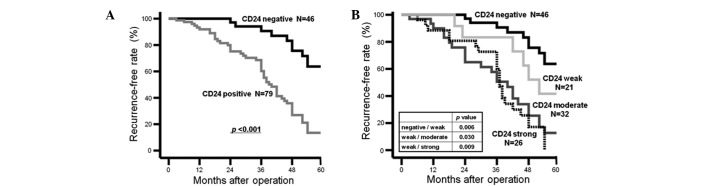
Analysis of the intra-bladder tumor recurrence following transurethral surgery performed using the Kaplan-Meier method. Kaplan-Meier curves of (A) the cancer recurrence-free rate in CD24-negative and -positive populations and (B) according to the intensity of CD24 staining. The statistical significance of any differences was calculated using a log-rank test. CD24, cluster of differentiation-24.

The recurrence-free rate of the bladder tumors following surgical treatment was analyzed using the Kaplan-Meier method. The recurrence-free rate was determined from the date of the TUR-Bt surgery to the time of the detection of intra-bladder cancer recurrence or the last follow-up. The 5-year overall recurrence-free rates of bladder cancer in the CD24-negative and -positive populations were 63.7 and 13.4%, respectively (Fig. 2A). The association between the intensity of CD24 expression and the recurrence-free rate was further analyzed during the follow-up period. There tended to be an association between the CD24 staining intensity and a poor recurrence-free rate (Fig. 2B). Log-rank tests revealed that there was a statistically significant difference between the negative/weak, weak/moderate and weak/strong staining categories.

**Figure 3.**
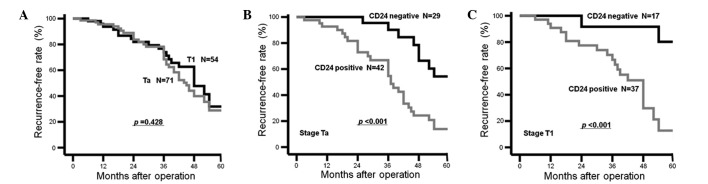
Analysis of intra-bladder tumor recurrence following treatment performed using the Kaplan-Meier method. Kaplan-Meier curves of (A) the cancer recurrence-free rates in the tumor stage Ta and T1 populations and according to the CD24 expression in (B) stage Ta and (C) stage T1. The statistical significance of any differences was calculated using log-rank tests. CD24, cluster of differentiation-24.

**Figure 4.**
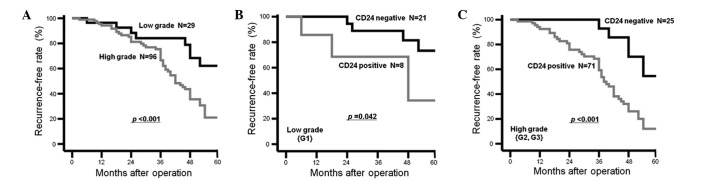
Analysis of intra-bladder tumor recurrence following treatment performed using the Kaplan-Meier method. Kaplan-Meier curves of (A) the cancer recurrence-free rates in the low (G1) and high (G2–G3) cancer grade populations and according to the CD24 expression in the (B) low and (C) high grade patients. The statistical significance of any differences was calculated using log-rank tests. CD24, cluster of differentiation-24.

The impact of the tumor stage and cancer grade on the recurrence-free rate was also investigated. The Ta and T1 tumor stages exhibited no significant association with the recurrence-free rate (Fig. 3A). By contrast, the higher cancer grades (G2–G3) had a significant correlation with a poor recurrence-free rate (Fig. 4A). A significant association between positive CD24 staining and the incidence of tumor recurrence was observed in each tumor category [stages Ta and T1, low (G1) and high grades (G2–G3); Figs. 3B and C, 4B and C].

## Discussion

Intra-bladder tumor recurrence is a common feature of human bladder cancer and a major clinical concern for patients following transurethral treatment. Several studies have suggested a clonal nature for the recurrent urothelial carcinomas and also that these tumors are often derived from disseminated cancer cells that remain following surgery (11–13). CD24 is considered to function as an adhesion molecule and is known to bind to P-selectin, a protein expressed on the surface of thrombin-activated platelets and endothelial cells (5,6), and to L1, a member of the immunoglobulin superfamily that is expressed on neural and lymphoid cells (3,6). It is hypothesized that CD24 is able to support the adhesion of neutrophils or monocytes to thrombin-activated platelets or activated endothelial cells (4–6). In addition, the interaction of cancer cells with P-selectin via CD24 may be an important adhesion pathway involved in cancer metastasis and progression (3–5). It is possible that CD24-expressing tumor cells may disseminate more easily as a result of their enhanced ability to attach to normal endothelial cells.

Accumulating evidence suggests that CD24 is a significant marker for cancer metastasis and prognosis (3–5,14). Studies have suggested that CD24 overexpression may contribute to the metastasis and subsequent poor prognosis of various metastatic tumors (3,4). Based on this background, the present study focused on the expression of CD24, a possible cancer cell dissemination-related factor, in human superficial bladder cancer and investigated its correlation with intra-bladder tumor recurrence following transurethral surgical treatment. Immunohistochemical analysis clearly indicated that there was a positive correlation between CD24 expression and the pathological cancer grade (low and high) that indicates the degree of malignancy. Positive CD24 expression was significantly associated with intra-bladder tumor recurrence following surgery. The association between CD24 expression and tumor recurrence was observed in each tumor category [stages Ta and T1, low- (G1) and high-grades (G2–G3)]. Notably, the intensity of CD24 expression was also correlated with the intra-bladder tumor recurrence following treatment for bladder cancer. This demonstrated that CD24 expression was significantly associated with the recurrence of bladder cancer. The significant association of CD24 expression with the cancer grade and tumor recurrence rate suggests that CD24 induced a more malignant phenotype in the bladder cancer cells and that it may be involved in tumor recurrence by enhancing the attachment of tumors cells to the normal endothelium.

The present study is the first to show the potential involvement of CD24 in the development of more malignant bladder cancer and in the recurrence of tumors. The results clearly indicated that higher CD24 protein expression may predict a higher risk of bladder cancer recurrence. CD24 expression may therefore be used not only as a prognostic marker in bladder cancer, but also as a target for the development of new therapeutic approaches, including antibody-based immunotherapeutic agents.

## Figures and Tables

**Table I.** Patient and bladder cancer characteristics.

Factor	Value
Number of patients (%)	125 (100)
Mean age (years)	61.4±1.4
Age range (years)	18–88
Gender (No., %)	
Male	97 (77.6)
Female	28 (22.4)
Stage classification (No., %)	
Ta	71 (56.8)
T1	54 (43.2)
Grade (No., %)	
G1	29 (23.2)
G2	62 (49.6)
G3	34 (27.2)
CD24 staining (No.)	
Negative	46 (36.8)
Positive	
Weak	21 (16.8)
Moderate	32 (25.6)
Strong	26 (20.8)
Recurrence (No., %)	
Negative	58 (46.4)
Positive	67 (53.6)
Mean follow-up (months)	34.7±1.6

G1, low-grade tumor; G2–3, high-grade tumors.

**Table II.** Association of CD24 expression with the clinicopathological parameters of bladder cancer.

Characteristics	Number (%)	Expression of CD24 protein		P-value
		Negative (%)	Positive (%)	
Total	125 (100)	46 (36.8)	79 (63.2)	0.27
Male	97 (77.6)	33 (34.0)	64 (66.0)	
Female	28 (22.4)	13 (46.4)	15 (53.6)	
Stage classification				0.35
Ta	71 (56.8)	29 (40.8)	42 (59.2)	
T1	54 (43.2)	17 (31.5)	37 (68.5)	
Grade				<0.001
Low (G1)	29 (23.2)	21 (72.4)	8 (27.6)	
High (G2, G3)	96 (76.8)	25 (26.0)	71 (74.0)	
Recurrence				<0.001
Negative	58 (46.4)	36 (62.1)	22 (37.9)	
Positive	67 (53.6)	10 (14.9)	57 (85.1)	

CD24, cluster of differentiation-24.

## References

[1] Jemal A, Siegel R, Xu J, Ward E (2010). Cancer statistics, 2010. CA Cancer J Clin.

[2] Kay R, Rosten PM, Humphries RK (1991). CD24, a signal transducer modulating B cell activation responses, is a very short peptide with a glycosyl phosphatidylinositol membrane anchor. J Immunol.

[3] Baumann P, Cremers N, Kroese F, Orend G, Chiquet-Ehrismann R, Uede T, Yagita H, Sleeman JP (2005). CD24 expression causes the acquisition of multiple cellular properties associated with tumor growth and metastasis. Cancer Res.

[4] Lim SC (2005). CD24 and human carcinoma: tumor biological aspects. Biomed Pharmacother.

[5] Aigner S, Sthoeger ZM, Fogel M, Weber E, Zarn J, Ruppert M, Zeller Y, Vestweber D, Stahel R, Sammar M, Altevogt P (1997). CD24, a mucin-type glycoprotein, is a ligand for P-selectin on human tumor cells. Blood.

[6] Sammar M, Aigner S, Altevogt P (1997). Heat-stable antigen (mouse CD24) in the brain: dual but distinct interaction with P-selectin and L1. Biochim Biophys Acta.

[7] Watanabe M, Kashiwakura Y, Huang P, Ochiai K, Futami J, Li SA, Takaoka M, Nasu Y, Sakaguchi M, Huh NH, Kumon H (2009). Immunological aspects of REIC/Dkk-3 in monocyte differentiation and tumor regression. Int J Oncol.

[8] Zhang K, Watanabe M, Kashiwakura Y, Li SA, Edamura K, Huang P, Yamaguchi K, Nasu Y, Kobayashi Y, Sakaguchi M, Ochiai K, Yamada H, Takei K, Ueki H, Huh NH, Li M, Kaku H, Na Y, Kumon H (2010). Expression pattern of REIC/Dkk-3 in various cell types and the implications of the soluble form in prostatic acinar development. Int J Oncol.

[9] Huang P, Chen J, Wang L, Na Y, Kaku H, Ueki H, Sasaki K, Yamaguchi K, Zhang K, Saika T, Nasu Y, Watanabe M, Kumon H (2012). Implications of transcriptional factor, OCT-4, in human bladder malignancy and tumor recurrence. Med Oncol.

[10] Huang HY, Shariat SF, Sun TT, Lepor H, Shapiro E, Hsieh JT, Ashfaq R, Lotan Y, Wu XR (2007). Persistent uroplakin expression in advanced urothelial carcinomas: implications in urothelial tumor progression and clinical outcome. Hum Pathol.

[11] Denzinger S, Mohren K, Knuechel R, Wild PJ, Burger M, Wieland WF, Hartmann A, Stoehr R (2006). Improved clonality analysis of multifocal bladder tumors by combination of histopathologic organ mapping, loss of heterozygosity, fluorescence in situ hybridization, and p53 analyses. Hum Pathol.

[12] Sidransky D, Frost P, Von Eschenbach A, Oyasu R, Preisinger AC, Vogelstein B (1992). Clonal origin bladder cancer. N Engl J Med.

[13] Junker K, Wolf M, Schubert J (2005). Molecular clonal analysis of recurrent bladder cancer. Oncol Rep.

[14] Overdevest JB, Knubel KH, Duex JE, Thomas S, Nitz MD, Harding MA, Smith SC, Frierson HF, Conaway M, Theodorescu D (2012). CD24 expression is important in male urothelial tumorigenesis and metastasis in mice and is androgen regulated. Proc Natl Acad Sci USA.

